# Two-dimensional dysprosium(III) coordination polymer: Structure, single-molecule magnetic behavior, proton conduction, and luminescence

**DOI:** 10.3389/fchem.2022.974914

**Published:** 2022-08-08

**Authors:** Jin-Fen Chen, Yi-Liang Ge, Dong-Hui Wu, Hao-Tian Cui, Zhi-Lin Mu, Hong-Ping Xiao, Xinhua Li, Jing-Yuan Ge

**Affiliations:** College of Chemistry and Materials Engineering, Wenzhou University, Wenzhou, China

**Keywords:** coordination polymer, dysprosium, slow magnetic relaxation, proton conduction, multifunctional

## Abstract

A new dysprosium (III) coordination polymer [Dy(Hm-dobdc) (H_2_O)_2_]·H_2_O (**Dy-CP**), was hydrothermal synthesized based on 4,6-dioxido-1,3-benzenedicarboxylate (H_4_m-dobdc) ligand containing carboxyl and phenolic hydroxyl groups. The Dy(III) center adopts an octa-coordinated [DyO_8_] geometry, which can be described as a twisted square antiprism (*D*_4d_ symmetry). Neighboring Dy(III) ions are interconnected by deprotonated Hm-dobdc^3−^ ligand to form the two-dimensional infinite layers, which are further linked to generate three-dimensional structure through abundant hydrogen bonds mediated primarily by coordinated and lattice H_2_O molecules. Magnetic studies demonstrates that **Dy-CP** shows the field-induced slow relaxation of magnetization and the energy barrier *U*_eff_/*k*_B_ and relaxation time *τ*_0_ are 35.3 K and 1.31 × 10^–6^ s, respectively. Following the vehicular mechanism, **Dy-CP** displays proton conductivity with σ equal to 7.77 × 10^–8^ S cm^−1^ at 353 K and 30%RH. Moreover, luminescence spectra reveal that H_4_m-dobdc can sensitize characteristic luminescence of Dy(III) ion. Herein, good magnetism, proton conduction, and luminescence are simultaneously achieved, and thus, **Dy-CP** is a potential multifunctional coordination polymer material.

## Introduction

Coordination polymers (CPs) have potential applications in gas storage/separation, catalysis, magnetism, and proton conduction due to their customizable compositions and variable structures (Yaghi et al., 2003; Kitagawa et al., 2004; Ferey, 2008; Zhu et al., 2018; Yuan et al., 2020; Cai et al., 2021; Chakraborty et al., 2021). In particular, CPs can integrate these multiple properties into the same molecular composite, which is an excellent platform for designing advanced multifunctional materials (Wang et al., 2017; Ge et al., 2019b; Ye et al., 2020; Zhou et al., 2020; Fan et al., 2021). In the field of molecular magnetism, Ln-CPs are of particular interest, enabling the production of magnetic materials with diverse properties, such as single-molecule magnets (SMMs) (Baldovi et al., 2014; Liu et al., 2016; Zhong et al., 2022). As we know, the magnetic anisotropy of metal ions plays a very important role in the construction of SMMs (Woodruff et al., 2013; Zhu et al., 2021). In this regard, lanthanide Dy(III) ion, may carry significant anisotropy because of its intrinsically large and unquenched orbital contribution to the magnetic moment (Ding et al., 2018; Parmar et al., 2021; Li et al., 2022). Goodwin and co-workers made a breakthrough in Dy(III)-based SMMs, reporting compound that exhibits a high effective energy barrier of 1,760 K (Goodwin et al., 2017). Therefore, we consider Dy(III) coordination compounds to be promising candidates for designing high-performance SMMs. Moreover, the high coordination number and flexible coordination geometry of Dy(III) ions can produce various interesting frameworks. Up to now, multifarious Dy-CPs with slow magnetic relaxation behaviors have been developed (Wu et al., 2020; Song et al., 2021; Su et al., 2021). Nevertheless, the inherent magnetisms of Dy(III) ions are very sensitive to various factors such as coordination geometry, magnetic interactions, etc., making the performance of Dy(III)-based SMMs difficult to predict (Pinkowicz et al., 2015; Zhang et al., 2015; Ge et al., 2020). More new topologies need to be established to study the magneto-structural correlations in depth.

Developing multifunctional magnetic CPs is currently a very attractive research topic, where magnetism can be integrated with other properties (such as proton conduction, sensing, or luminescence) to achieve multi-task expression and expand the application range of materials (Chen et al., 2017; Bera et al., 2018; Minguez Espallargas and Coronado, 2018). Among them, proton-conducting materials are potential replacements for Nafion ionomers in the catalyst layer of fuel cells, which can produce environmentally friendly energy (Yamada et al., 2013; Ramaswamy et al., 2014; Meng et al., 2017; Li et al., 2020). The easily tunable crystal structures and modifiable pore environment of CPs are ideal crystal models for designing proton conductors and gaining insight into proton transfer mechanisms (Su et al., 2020). Studies have shown that designing and developing complex hydrogen bond networks is one of the efficient strategies to improve proton conductivity in CPs, such as introducing functional Brønsted acid groups (-COOH and -OH) (Biswas et al., 2017; Bera et al., 2018).

Based on the above considerations, we envisioned that combining Dy(III) ion with carboxyl- and hydroxyl-rich organic ligand would be a sensible strategy to engineer SMM behavior with proton conduction into functional CPs. We chose the aromatic ligand 4,6-dioxido-1,3-benzenedicarboxylate (H_4_m-dobdc), and to our knowledge, Dy(III) complexes based on this ligand have been not been reported (Kapelewski et al., 2018; Barnett et al., 2019). Carboxyl and phenolic hydroxyl groups have high affinity with Dy(III) ion and diverse coordination modes, and more importantly, they can also act as efficient hydrogen bond acceptors/donors, forming infinite hydrogen bond networks to facilitate proton transport (Li et al., 2017; Wang et al., 2021; Bhadra et al., 2022). Herein, a two-dimensional (2D) CP [Dy(Hm-dobdc) (H_2_O)_2_]·H_2_O (**Dy-CP**), was hydrothermal synthesized through the interaction of Dy(III) ion and judiciously selected organic ligands, and its field-induced slow relaxation behavior and proton conduction properties were demonstrated.

## Experimental sections

### Synthesis of [Dy(Hm-dobdc) (H_2_O)_2_]·H_2_O (Dy-CP)

The reactants H_4_m-dobdc (0.0297 g, 0.15 mmol), Dy(NO_3_)_3_·6H_2_O (0.0918 g, 0.2 mmol), and 10 ml H_2_O were placed in a 15 ml Teflon cup. The mixture was heated to 140°C for 3 days. After cooling, the light brown block crystals of **Dy-CP** were obtained with a yield of 24% (based on H_4_m-dobdc). Anal. Calcd (%): C, 23.34; H, 2.20%. Found: C, 23.18; H, 2.14%. IR (cm^−1^, KBr): 3,859.56(s), 3,468.01(w), 3,217.27(w), 2,657.91(w), 1,853.59(w), 1,811.16(w), 1,780.3(w), 1,720.5(s), 1,705.07(s), 1,643.35(s), 1,566.2(m), 1,519.91(w), 1,465.9(m), 1,400.32(s), 1,346.31(m), 1,301.95(m), 1,226.73(w), 1,195.87(s), 1,083.99(w), 956.69(w), 893.04(m), 844.82(m), 819.75(w), 783.1(w), 754.17(w), 723.31(m), 700.16(m), 677.01(w), 653.87(m), 619.15(m), 578.64(w), 526.57(w), 472.56(m), 430.13(w).

## Result and disscussion

### Description of crystal structure

**FIGURE 1:**
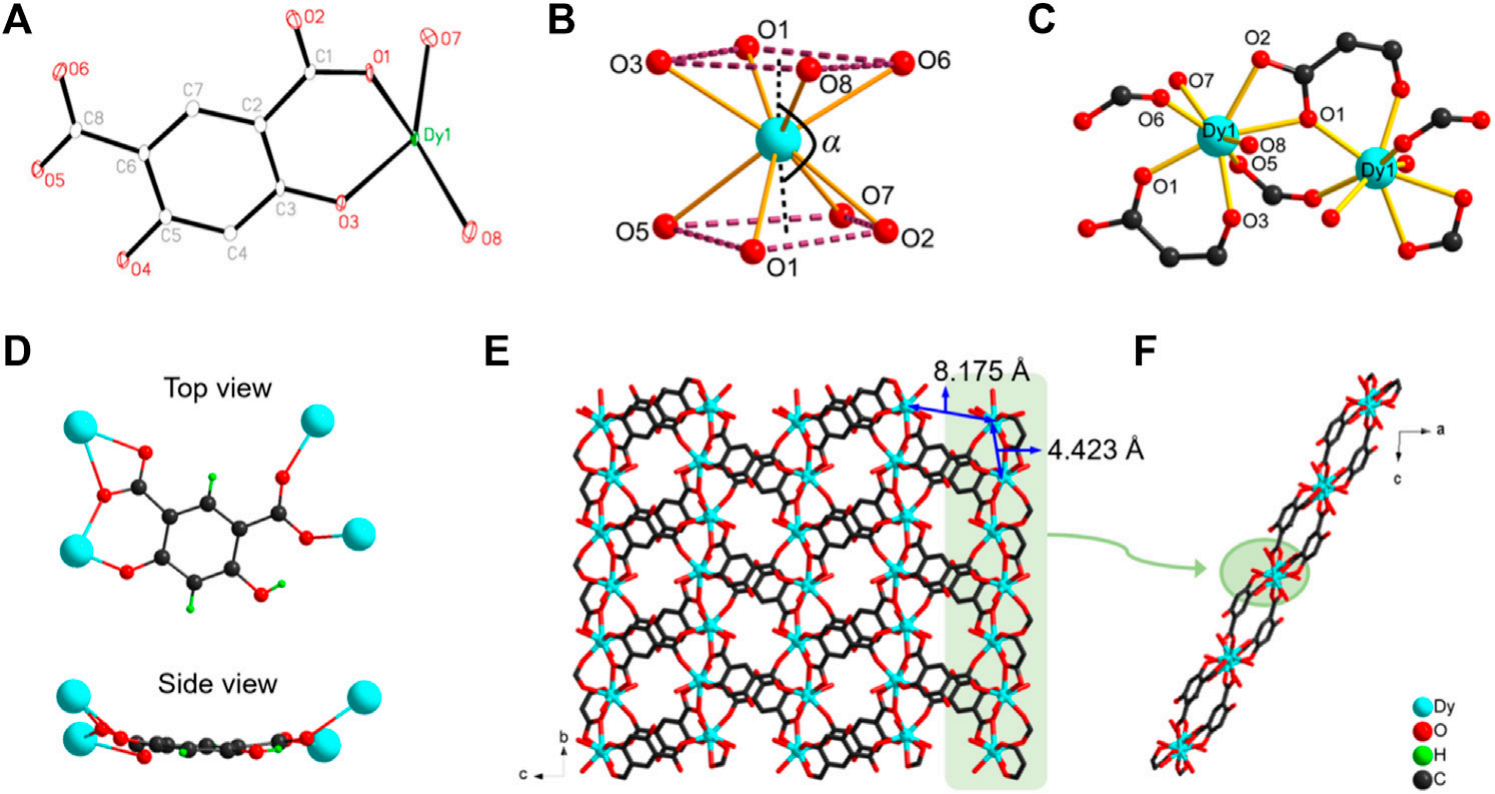
**(A)** The labeled asymmetric unit of **Dy-CP (B)** coordination sphere of Dy1 center **(C)** connection between adjacent metal centers **(D)** top view and side view of coordination mode of Hm-dobdc^3−^ linker **(E)** 2D structure of **Dy-CP** in the *bc* plane **(F)** side view of the 2D layer. Uncoordinated H_2_O molecules are removed for clarity.

The brown-orange block crystals of [Dy(Hm-dobdc) (H_2_O)_2_]·H_2_O **(Dy-CP**) were obtained by the reaction of H_4_m-dobdc and Dy(NO_3_)_3_·6H_2_O at 140°C. Single-crystal analysis shows that **Dy-CP** crystallizes in the monoclinic space group *P*2_1_/n, and the crystallographic data are summarized in Supplementary Table S1. Its asymmetric unit involves one Dy(III) ion, one Hm-dobdc^3−^ ligand, two coordinated H_2_O molecules, and one uncoordinated H_2_O molecule (Figure 1A). The Dy1 center adopts an octa-coordinated [DyO_8_] geometry with four O_carboxylate_ atoms (O1, O2, O5, and O6) from three Hm-dobdc^3−^ ligands, the another O_carboxylate_ atom (O1) and one O_phenoxide_ atom (O3) from one Hm-dobdc^3−^ ligand, and two O_water_ atom (O7 and O8) from two coordinated H_2_O (Figure 1B and Supplementary Figure S1). Dy−O bond lengths are in the range of 2.287 (3) Å to 2.495 (3) Å, similar to those reported for Dy(III) compounds with oxygen donors (Supplementary Table S2) (Song et al., 2021). Shape analysis revealed that the exact geometry of Dy1 ion can be assigned to a twisted square antiprism (*D*_4d_ symmetry) with a SAPR-8 factor of 1.244 (Supplementary Table S3) (Ge et al., 2017). As shown in Figure 1B, the Dy1 ion is unevenly distributed between two square planes. The distance of Dy1 ion from the center of the top plane (O1, O3, O6 and O8) is 1.225 Å, which is closer than the distance (1.415 Å) to the center of the bottom plane (O1, O2, O5 and O7). The dihedral angle between these two planes is 5.157°, and the bending angle *α* defined as center-Dy1-center is 171.45°.

One Hm-dobdc^3−^ ligand is coordinated to four Dy1 ions *via* one deprotonated phenolic hydroxyl group and two deprotonated carboxyl groups. Of the two carboxyl groups, one is ligated in a *μ*_2_-*η* ^2^:*η* ^1^ chelating mode and the other in a *μ*_2_-*η* ^1^:*η* ^1^ mode (Figure 1D). After coordination, the Hm-dobdc^3−^ is not planar viewed from the side. The multiple coordination sites and variable coordination configuration of Hm-dobdc^3−^ play a key role in constructing **Dy-CP**. The adjacent Dy1 ions are linked together by one *μ*_2_-*η* ^1^:*η* ^1^ carboxylate group and one *μ*_*2*_-O1 atom from Hm-dobdc^3−^ to generate the one-dimensional metal chain along the crystallographic *b*-axis (Figures 1C–E). The nearest Dy···Dy separation is 4.423 Å and Dy1-O1-Dy1 angle is 132.85°. Each chain is linked by the polytopic Hm-dobdc^3−^ ligand (Dy···Dy = 8.175 Å) generating the 2D infinite layer (Figures 1E,F).

**FIGURE 2:**
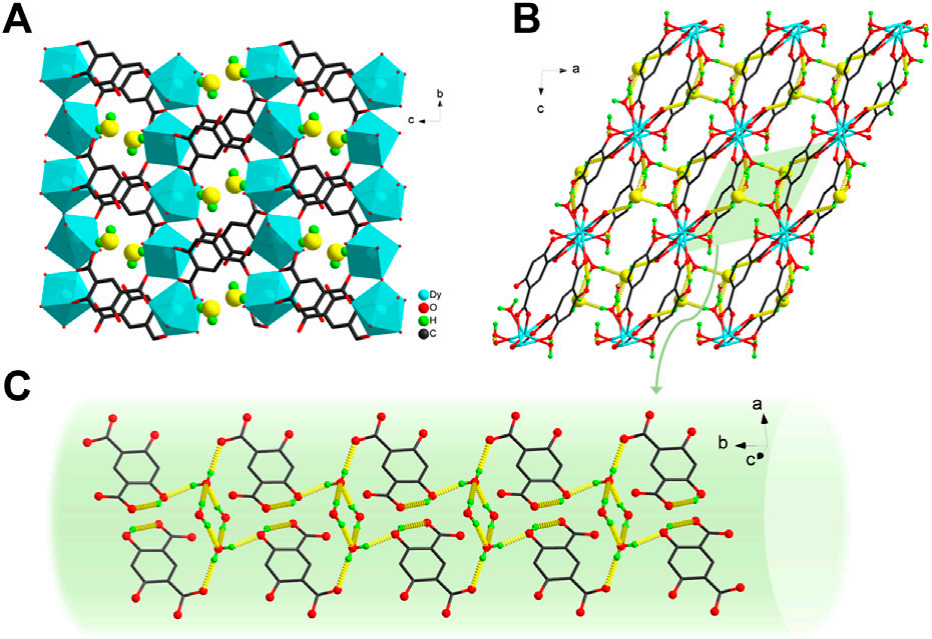
**(A)** The stacking motif of **Dy-CP** along the crystallographic *a*-axis. Yellow balls represent the O atoms of lattice H_2_O molecules **(B)** 3D framework driven by the O−H···O hydrogen bonds (yellow dashed lines) **(C)** an enlarged view of the hydrogen bonds formed between lattice H_2_O molecule, coordinated H_2_O molecule and Hm-dobdc^3−^ ligands. Some unrelated atoms are omitted for clarity.

In the stacking motif, these 2D layers are stacked along the crystallographic *a*-axis in an–AAA–fashion, generating small-sized pores (Figure 2A). Furthermore, uncoordinated phenolic hydroxyl group is oriented towards the interior of the pore to create a targeted hydrophilic environment in which the uncoordinated H_2_O molecules reside. Abundant O−H···O hydrogen bonds are formed between the lattice H_2_O molecule, coordinated H_2_O molecule and the Hm-dobdc^3−^ ligand (Supplementary Table S4) (Wang et al., 2009). One coordinated H_2_O molecule (O8) and one lattice H_2_O molecule (O9) and their symmetry-related counterparts yield a centrosymmetric cyclic H_2_O tetramer (Supplementary Figure S2). In the tetramer, the O8 water monomer is the hydrogen bond donor and the O9 atom acts as the acceptor. The average distance of O···O is only 2.727 Å. This hydrogen-bonding network is beneficial for stabilizing H_2_O molecules (Sasaki et al., 2018). The tetramers link the adjacent layers to generate a 3D framework (Figures 2B,C). The hydrophilicity and multiple hydrogen bonds facilitate the exploration of proton conduction (Meng et al., 2017).

### FT-IR spectra, purity and structural stability

**FIGURE 3:**
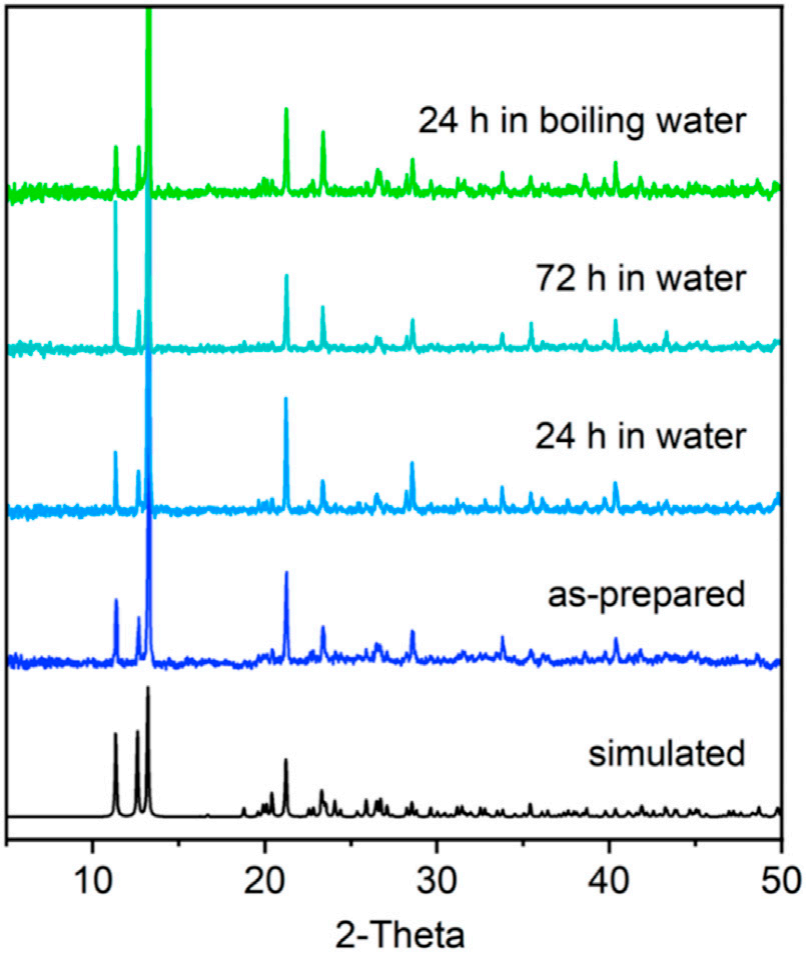
PXRD patterns of synthesized **Dy-CP** (5–50°) sample and those were treated with water for several days.

The FTIR spectra of H_4_m-dobdc and **Dy-CP** are shown in Supplementary Figure S3. Both samples contain a broad -OH stretching vibration absorption band around 3,200 cm^−1^. Compared with the free ligand, the shift of the characteristic peaks for the symmetric and asymmetric stretching of the carboxyl groups in **Dy-CP** suggests that H_4_m-dobdc reacts with Dy(III) site. The enhanced absorption band in the 3,300–3,700 cm^−1^ region in **Dy-CP** indicates the presence of H_2_O molecules directly coordinating to the Dy(III) sites and/or generating hydrogen bonds (Vitillo and Ricchiardi, 2017). Thermogravimetric analysis (TGA) curve reveals that the lattice H_2_O molecule can be stored in the pore of **Dy-CP** at room temperature and higher, with release occurring around 90–192°C (weight loss of 4.40%, calculated 4.37%, Supplementary Figure S4). Moreover, maintaining better stability in aqueous solution is a prerequisite for CPs to be used as proton-conducting materials (Yuan et al., 2018; Su et al., 2020; Yang et al., 2021). Powder X-ray diffraction (PXRD) measurement confirms the absence of any other phases in **Dy-CP**, with the experimental diffraction peak positions consistent with that simulated using crystal data (Figure 3). The synthesized samples were immersed in water and boiling water for several days. PXRD profiles of all water-soaked samples are in good agreement with the pristine one, indicating the retained crystallinity of **Dy-CP** in water (Figure 3). The good stability in water will provide new opportunity for proton conduction.

### Magnetic properties

**FIGURE 4:**
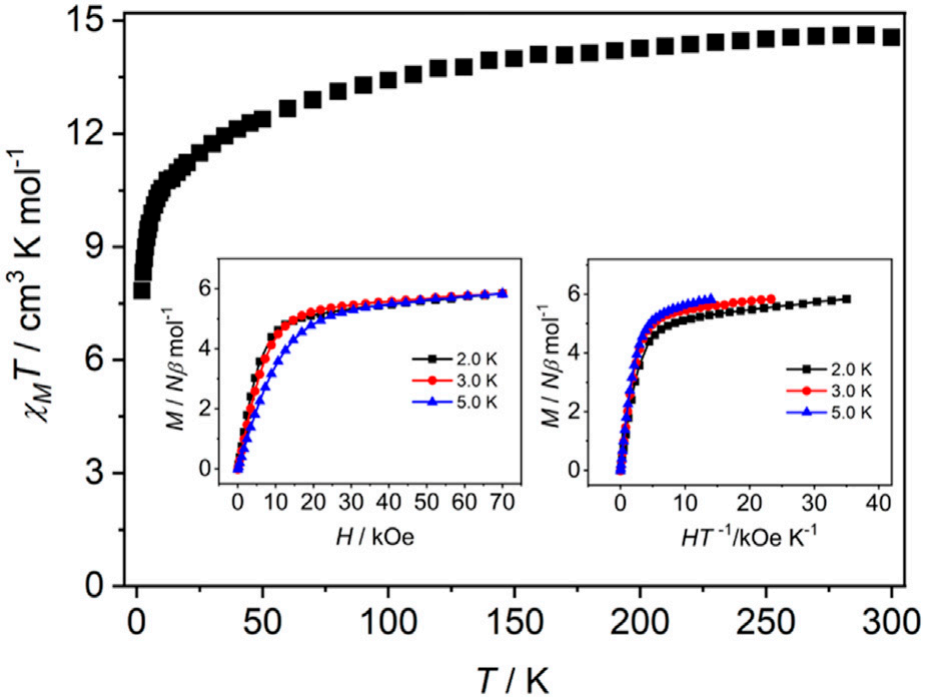
Temperature-dependent *χ*_M_ *T* for **Dy-CP**. The insets represent *M*-*H* and *M*-*H T* ^−1^ plots.

The direct-current (DC) magnetic susceptibilities experiments were carried out on polycrystalline samples of **Dy-CP** in an applied field of 1 kOe between 2 and 300 K (Figure 4). The *χ*_M_ *T* value at 300 K is 14.56 cm^3^ K mol^−1^, which is a little higher than the theoretical value for a free Dy(III) ion (14.17 cm^3^ K mol^−1^; *g* = 4/3, *J* = 15/2) (Cui et al., 2021). With a lowering of the temperature from 300 to 10 K, the *χ*_M_ *T* value decreases gradually, and then drops rapidly to the minima of 7.84 cm^3^ K mol^−1^ at 2 K, which may be caused by the antiferromagnetic interactions between adjacent Dy(III) ions and/or the progressive depopulation of the excited Stark sublevels of Dy(III) ions (Wu et al., 2020). Considering the slightly longer Dy⋅⋅⋅Dy distance compared to the literature reports, antiferromagnetic interaction maybe not dominate in **Dy-CP**.

The field-dependent magnetization (*M*) of **Dy-CP** was also collected in the field (*H*) range of 0–70 kOe at 2.0, 3.0 and 5.0 K, respectively (Figure 4 inset). The *M* value of **Dy-CP** increases slowly as *H* increases, and a maximum of 5.84 *Nβ* is reached at 70 kOe and 2.0 K. The nonsaturation of *M* and the non-superimposed isothermal magnetization curves (*M* vs. *H T* ^−1^) suggest the presence of low-lying excited states and/or significant magnetic anisotropy in **Dy-CP** (Ge et al., 2019a; Cui et al., 2021).

**FIGURE 5:**
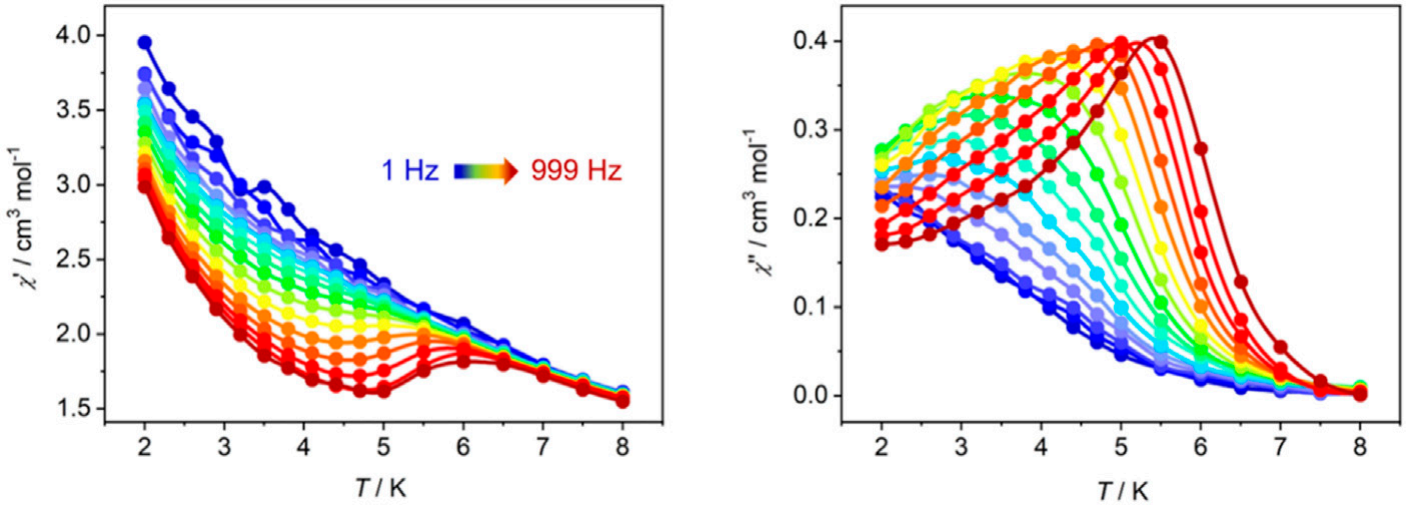
Temperature-dependent *χ*′ (left) and *χ*″ (right) ac susceptibilities for **Dy-CP** measured in 1.5 kOe dc field.

**FIGURE 6:**
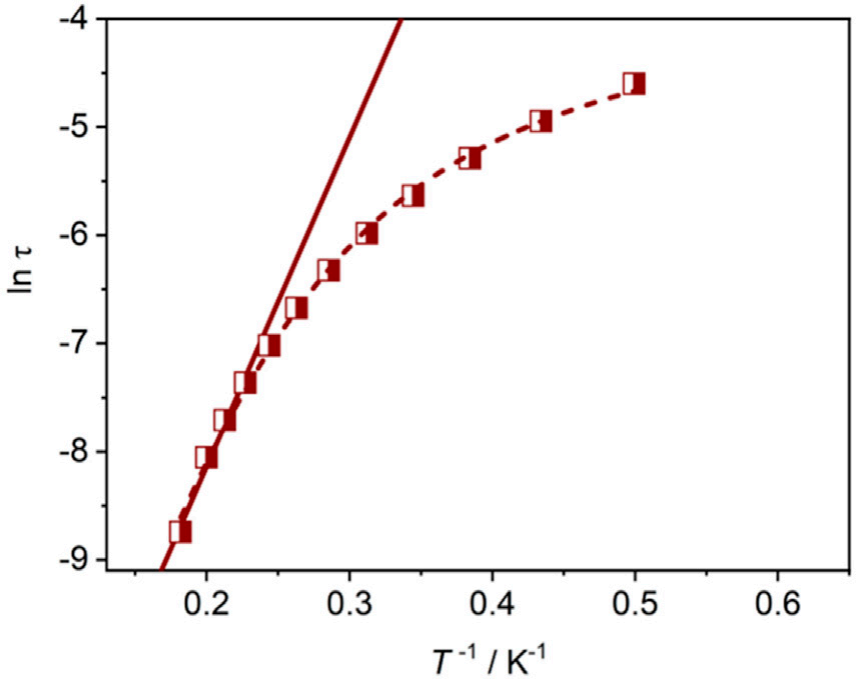
ln τ vs. T^−1^ plot for **Dy-CP** and the Arrhenius law *τ* ^−1^ = *τ*_0_^−1^ exp (-*U*_eff_ */k*_B_T) (solid) and Equation *τ* ^−1^ = *AH* ^2^ *T* + *CT* ^n^ + *τ*_0_^−1^exp (-*U*_eff_ */k*_B_ *T*) (dashed) fitting lines.

Considering the magnetic anisotropy of Dy(III) ion, the alternating-current (ac) magnetic susceptibilities of **Dy-CP** were measured to explore the dynamic magnetic behavior. Under zero dc field, the out-of-phase (*χ*″) signals keep silent at high frequency of 707 Hz (Supplementary Figure S5). When an additional 1.5 kOe dc field is applied, the good-shaped peaks can be easily observed in the *χ*″ vs. *T* graph (Figure 5). The peak position of the *χ*"(*T*) signal shifts gradually to the high temperature component as the frequency increases, showing the obvious slow magnetic relaxation expected for SMMs (Chen et al., 2016). At 999 Hz, the maximum value of *χ*"(*T*) appears around 5.5 K. The relaxation time *τ* was extracted from the peaks of *χ*″ signals in Figure 5A. At the high temperature, *τ* is linearly dependent on *T* ^−1^, which can be fitted using Arrhenius law to afford the thermal energy barrier (*U*_eff_ */k*_B_) and the pre-exponential factor (*τ*_0_) are 30.3 K and 6.82 × 10^–7^ s, respectively (Figure 6), confirming a field-induced SMM performance (10^–6^–10^–11^ s) (Bera et al., 2018; Bera et al., 2019). At the lower temperature, the relationship between ln *τ* and *T* ^−1^ deviates from the linearity of Arrhenius law, suggesting the intervention of other possible relaxation processes.

For further investigation of the magnetic dynamics, the frequency-dependent ac susceptibilities were also collected. *χ*"(*F*) peaks can be observed clearly in the high frequency region, as shown in Supplementary Figure S6. Slightly higher *χ*" values in the low frequency region, especially at lower temperatures, suggest that other relaxation processes may exist (Gonzalez et al., 2021). Its Cole−Cole diagrams exhibit semicircular shape in the high frequency and irregular shape in the low frequency regions. Fitting the data between 2.3 and 5.5 K by the extended Debye functions (Gao et al., 2018) gives *α* value ranging from 0.05 to 0.16 for the relaxation in high frequency region (Supplementary Figure S7). Unfortunately, the fit for the low frequency region is unsuccessful due to limited frequency and/or temperature. The fit of *τ* takes into account the multiple relaxation processes reveals that the relaxation occurs *via* the temperature-dependent Orbach (*τ*_0_^−1^exp (-*U*_eff_ */k*_B_ *T*)), Raman (*CT* ^n^), and direct (*AH* ^2^ *T*) mechanisms (Figure 6 short dashed line). Parameters *A* = 1.93 × 10^–5^ s^−2^ Oe^−2^, *n* = 5.27, *C* = 0.50 s^−1^ K^−5.27^, *U*_eff_ */k*_B_ = 35.3 K, and *τ*_0_ = 1.31 × 10^–6^ s were obtained, which are consistent with the expectations of Kramer ion Dy(III)-based SMMs (Ge et al., 2017).

### Proton conduction

**FIGURE 7:**
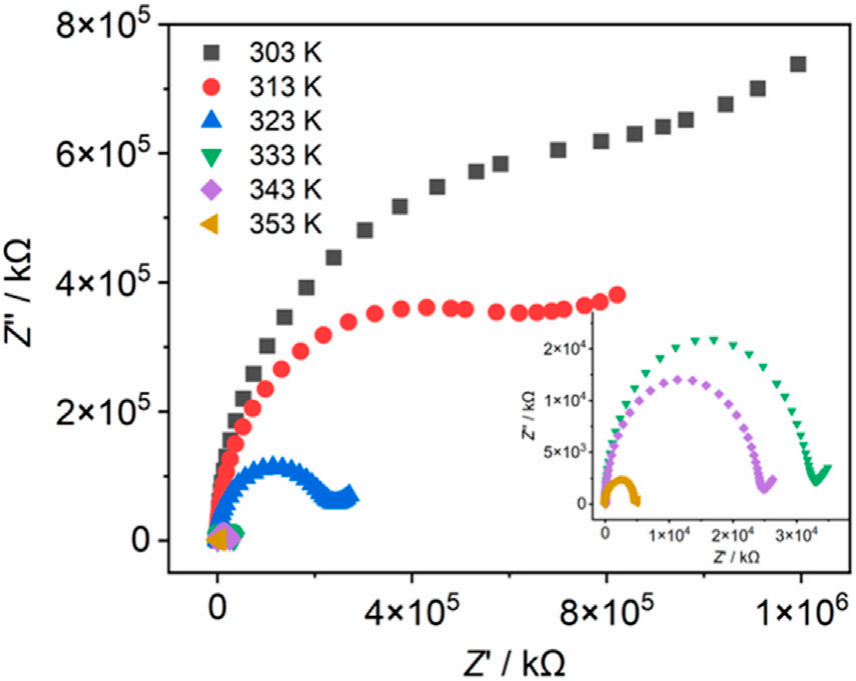
Nyquist plots of **Dy-CP** measured at 30% RH and indicated temperatures.

The presence of an intricate network of hydrogen bonds in **Dy-CP** suggests the efficient proton transfer pathways. Subsequent ac impedance of the compact pellet was measured under controlled experimental condition, and the proton conductivities (σ) were calculated by fitting the Nyquist plots. At 303 K and 30% relative humidity (RH), the Nyquist plot displays a partial semicircle in the high frequency component and a small oblique tail in the low frequency component, which is the fingerprint of proton transport behavior (Elahi et al., 2019). The related σ is 4.37 × 10^–10^ S cm^−1^ (Figure 7). Further studies found that the proton conductivity of **Dy-CP** is temperature dependent. As temperature increases, the size of the semicircle appears to decrease significantly, corresponding to the enhanced conductivity. At 353 K, σ reaches 7.77 × 10^–8^ S cm^−1^. This trend in conductivity can be explained by several plausible reasons, (i) the pK_w_ values of the coordinated and lattice H_2_O molecules decrease, favoring the release of proton; (ii) the stable existence of lattice H_2_O molecules at elevated temperature, facilitating the preservation of strong hydrogen bonds and (iii) thermally assisted proton hopping on hydrogen-bonding array containing H_2_O molecules (Tang et al., 2014; Bera et al., 2018). According to the linear fit of Arrhenius law σT = σ_0_ exp (*E*_a_/k_B_T), the activation energy *E*_a_ = 0.93 eV is estimated (Supplementary Figure S8). Value more than 0.4 eV indicates that a vehicular mechanism operates for proton conduction in **Dy-CP** (Su et al., 2020). Additionally, the structural of **Dy-CP** was integrated after the impedance measurement, as PXRD pattern demonstrated (Supplementary Figure S9).

### Luminescence property

The solid-state luminescence property of **Dy-CP** was measured at room temperature. When excited at 329 nm, **Dy-CP** exhibits two emission peaks at 481 and 475 nm, corresponding to hypersensitive ^4^F_9/2_–^6^H_15/2_ and ^4^F_9/2_–^6^H_13/2_ transitions of Dy(III) ion (Supplementary Figure S10). Notably, the disappearance of the broadband emission of the ligand implies an effective energy transfer from the ligand to the metal, and H_4_m-dobdc ligand brings an efficient antenna effect (Zhong et al., 2020).

## Conclusion

In summary, a two-dimensional coordination polymer [Dy(Hm-dobdc) (H_2_O)_2_]·H_2_O (**Dy-CP**) containing abundant hydrogen bonds has been successfully prepared and structurally characterized. Magnetic investigation demonstrates that **Dy-CP** exhibits the field-induced SMM property with the energy barrier equal to 35.3 K. The impedance analysis of **Dy-CP** displays proton conductivity (7.77 × 10^–8^ S cm^−1^ at 353 K) at 30% RH. Furthermore, luminescence spectra reveal that H_4_m-dobdc can sensitize characteristic luminescence of Dy(III) ion at 481 and 475 nm. This phenomenon suggests that introducing Dy(III) ion and functional carboxyl and phenolic hydroxyl groups is beneficial for the development of multifunctional coordination polymers possessing luminescence, proton conduction, and magnetism.

## Data Availability

The datasets presented in this study can be found in online repositories. The names of the repository/repositories and accession number(s) can be found in the article/Supplementary Material.
