# Supplementary material for: Two-dimensional dysprosium(III) coordination polymer: Structure, single-molecule magnetic behavior, proton conduction, and luminescence
Source: Front Chem. 2022 Aug 8;10:974914. doi: 10.3389/fchem.2022.974914 (PMC9393541; doi:10.3389/fchem.2022.974914)
Supplement: Supplementary file 1 [file DataSheet1.docx]

Supplementary Material for

**Two-Dimensional Dysprosium(III) Coordination Polymer: Structure, Single-Molecule Magnetic Behavior, Proton Conduction, and Luminescence**

**Jin-Fen Chen^1^, Yi-Liang Ge^1^, Dong-Hui Wu^1^, Hao-Tian Cui^1^, Zhi-Lin Mu^1^, Hong-Ping Xiao^1*^, Xinhua Li^1*^, and Jing-Yuan Ge^1*^**

^1^College of Chemistry and Materials Engineering, Wenzhou University, Wenzhou 325035, P. R. China

.**Contents**

**Materials and physical measurements**

**X-ray Crystallography**

**Supplementary Table S1.** Crystallographic data for **Dy-CP.**

**Supplementary Table S2.** Selected bond lengths (Å) and bond angles (°) around Dy(III) center in **Dy-CP.**

**Supplementary Table S3**. Shape analysis for the Dy(III) centers of compound **Dy-CP**.

**Supplementary Table S4.** Summary of hydrogen bonds in **Dy-CP**.

**Supplementary Figure S1.** One Dy(III) center connected by four Hm-dobdc^3−^ ligand and two water molecules.

**Supplementary Figure S2.** The coordination environment of the water tetramer located between neighboring layers of **Dy-CP** (lift), and basic hydrogen-bonding building block of tetramer unit (right).

**Supplementary Figure S3.** The FT-IR spectra of H_4_m-dobdc and **Dy-CP**.

**Supplementary Figure S4.** Thermogravimetric analysis (TGA) curve of **Dy-CP.**

**Supplementary Figure S5.** Temperature-dependent in-phase *χ*′ (up) and out-of-phase *χ*″ (blow) ac susceptibilities for **Dy-MOF** measured under zero dc field with an ac frequency of 707 Hz.

**Supplementary Figure S6.** Frequency-dependent *χ*′ (left) and *χ*″ (right) ac susceptibilities for **Dy-CP** measured in 1.5 kOe dc field.

**Supplementary Figure S7.** Cole–Cole plots for **Dy-MOF** obtained using the ac susceptibility data at a 1.5 kOe dc field. The solid lines represent the fit to the extended Debye model.

**Supplementary Figure S8.** Plot of ln (*σT*) versus 1000*T*^-1^, where the solid red line represents the fitted curve based on the Einstein-Nernst equation, revealing the value of activation energy (*E*_a_)

**Supplementary Figure S9.** PXRD patterns of **Dy-CP** for the simulated, synthesized, and sample after proton conduction measurement.

**Supplementary Figure S10.** The solid-state excitation and emission spectra of H_4_m-dobdc (left) and **Dy-CP** (right) at room temperature.

**Supplementary References**

**Materials and physical measurements**

All starting chemicals and solvents were commercially available and used directly without further purification. FT-IR spectra were measured on a Fourier transform infrared spectrometer (IRAffinity-1S) as KBr pellets in the 400-4000 cm^−1^ region. Elemental analysis for C and H was performed on a Perkin-Elmer 240C analyzer. Experimental powder X-ray diffraction (PXRD) data were collected using a Bruker D8 Advance diffractometer with Cu-Kα radiation (*λ* = 1.5418 Å) at room temperature in the 5–50°region. The simulated PXRD spectra were generated using the program Mercury 3.8. Solid-state luminescent spectra were recorded on a F-4700 spectrofluorometer at room temperature with a light source of Xenon lamp.

Magnetic susceptibilities were measured using a Quantum Design MPMS-SQUID-VSM magnetometer. The variable-temperature magnetic susceptibilities were performed in the range of 2–300 K. Field-dependent magnetizations were collected with external direct current (dc) field ranging from 0 to 70 kOe. For dynamic magnetic susceptibilities, the oscillating alternating current (ac) field is 2 Oe. The diamagnetic contributions of the sample holder and constituent atoms were estimated using Pascal’s constants (S1).

Proton conductivity was performed on a CHI604E electrochemical workstation by four-electrode voltage compensation method (S2). During the test, the sample was put into a self-made mold with a radius of 0.3 cm to afford circular pellets. Thickness was measured with vernier caliper. Silver colloid was then coated on two sides of the pellets and fixed on the sample stage with gold wires. The proton conductivities were measured on the pellets with a signal amplitude of 0.2 V in the frequency range from 1 Hz to 1 MHz. The measurements for **Dy-MOF** were operated at 30% relative humidity and normal temperatures 303 K, 313 K, 323 K, 333 K, 343 K and 353 K). The proton conductivity σ was calculated using the formula σ = *l* / (SR), where σ is the conductivity (S cm^–1^), *l* is the thickness (cm) of the pellet, *S* is the crosssectional area (cm^2^) of the pellet and *R* is the bulk resistance (Ω).

**X**-**ray** **Crystallography**

The single crystals of **Dy-MOF** were determined using a Bruker Smart-APEXII CCD diffractometer with Mo K*α* radiation (*λ* = 0.71073 Å) at 296(2) K for. The raw frame data were integrated into SHELX-format reflection files and corrected for Lorentz and polarization effects using SAINT software (S3). Corrections for incident and diffracted beam absorption effects were applied using SADABS supplied by Bruker (S4). The structure was solved and refined against *F*^2^ by the full-matrix least-squares using the SHELXL-2018/6 package (S5). The positions of the metal atoms and their first coordination spheres were located from direct method E-maps. All the non-hydrogen atoms were calculated theoretically and refined anisotropic. Hydrogen atoms were generated onto the specific atoms and refined isotropically with fixed thermal factors.

**Supplementary Table S1.** Crystallographic data for **Dy-CP**.

| Compound | **Dy-CP** |
| --- | --- |
| Empirical formula | C_8_H_9_O_9_Dy |
| Formula weight | 411.65 |
| Crystal system | Monoclinic |
| Space group | P2_1_/n |
| Temperature | 296 K |
| *a* (Å) | 7.646(2) |
| *b* (Å) | 8.698(3) |
| *c* (Å) | 15.607(5) |
| *α* (°) | 90.00 |
| *β* (°) | 93.360(5) |
| *γ* (°) | 90.00 |
| V (Å^3^) | 1036.1 |
| *ρ* calc (g/cm^3^) | 2.639 |
| *F*(000) | 780 |
| GOF on *F*^2^ | 1.090 |
| *R*_1_*^a^*, *wR*_2_*^b^* [*I*>2*σ*(I)] | 0.023, 0.062 |
| CCDC number | 2180230 |

**Supplementary Table S2.** Selected bond lengths (Å) and bond angles (°) around Dy(III) center in **Dy-CP**.

| Bond lengths (Å) | | | |
| --- | --- | --- | --- |
| Dy1-O1 | 2.331(2) | Dy1-O6^i^ | 2.287(3) |
| Dy1-O3 | 2.287(3) | Dy1-O5^ii^ | 2.367(3) |
| Dy1-O7 | 2.390(3) | Dy1-O1^iii^ | 2.495(3) |
| Dy1-O8 | 2.341(3) | Dy1-O2^iii^ | 2.443(3) |
| Bond angles (°) | | | |
| O6^i^-Dy1-O3 | 117.14(11) | O5^ii^-Dy1-O7 | 74.45(10) |
| O6i-Dy1-O1 | 73.34(9) | O6^i^-Dy1-O2^iii^ | 79.59(10) |
| O3-Dy1-O1 | 73.71(9) | O3-Dy1-O2^iii^ | 136.45(10) |
| O6^i^-Dy1-O8 | 76.11(12) | O1-Dy1-O2^iii^ | 147.40(9) |
| O3-Dy1-O8 | 71.56(10) | O8-Dy1-O2^iii^ | 74.80(12) |
| O1-Dy1-O8 | 114.75(11) | O5^ii^-Dy1-O2^iii^ | 110.04(11) |
| O6^i^-Dy1-O5^ii^ | 146.34(10) | O7-Dy1-O2^iii^ | 78.02(11) |
| O3-Dy1-O5^ii^ | 78.81(9) | O6^i^-Dy1-O1^iii^ | 128.53(9) |
| O1-Dy1-O5^ii^ | 84.51(9) | O3-Dy1-O1^iii^ | 92.45(9) |
| O8-Dy1-O5^ii^ | 137.17(11) | O1-Dy1-O1^iii^ | 158.12(4) |
| O6^i^-Dy1-O7 | 76.41(11) | O8-Dy1-O1^iii^ | 75.05(11) |
| O3-Dy1-O7 | 142.70(9) | O5^ii^-Dy1-O1^iii^ | 76.08(9) |
| O1-Dy1-O7 | 78.24(10) | O7-Dy1-O1^iii^ | 105.59(10) |
| O8-Dy1-O7 | 144.19(10) | O2^iii^-Dy1-O1^iii^ | 52.12(8) |

Symmetry codes: (i) *x*-1/2, -*y*+1/2, *z*+1/2; (ii) -*x*+1, -*y*+1, -*z*+1; (iii) -*x*+1/2, *y*+1/2, -*z*+3/2.

**Supplementary Table S3**. Shape analysis for the Dy(III) centers of compound **Dy-CP**.

| LnL8 | Shape | Symmetry | Dy1 |
| --- | --- | --- | --- |
| SAPR-8 | Square antiprism | *D*_4d_ | **1.244** |
| TDD-8 | Triangular dodecahedron | *D*_2d_ | 2.939 |
| JBTPR-8 | Biaugmented trigonal prism J50 | *C*_2v_ | 3.615 |
| BTPR-8 | Biaugmented trigonal prism | *C*_2v_ | 2.978 |

**Supplementary Table S4.** Summary of hydrogen bonds in **Dy-CP**.

| *D*−H···*A* | *D*−H | H···*A* | *D*···*A* | ∠*D*−H···*A* |
| --- | --- | --- | --- | --- |
| O9−H9B···O4^vi^ | 0.85 | 1.928 | 2.750 (4) | 162 |
| O9−H9A···O2^vii^ | 0.85 | 2.071 | 2.742 (4) | 135 |
| O8−H8B···O9^viii^ | 0.85 | 1.934 | 2.761 (4) | 164 |
| O8−H8A···O9 | 0.85 | 1.884 | 2.693 (5) | 158 |
| O7−H7B···O3^iv^ | 0.85 | 1.917 | 2.736 (4) | 161 |
| O4−H4···O5 | 0.82 | 1.850 | 2.579 (4) | 148 |

Symmetry codes: (iv) -*x*+1/2, *y*-1/2, -*z*+3/2; (vi) -*x*+3/2, *y*-1/2, -*z*+3/2; (vii) -*x*+3/2, *y*+1/2, -*z*+3/2; (viii) -*x*+1, -*y*+1, -*z*+2.


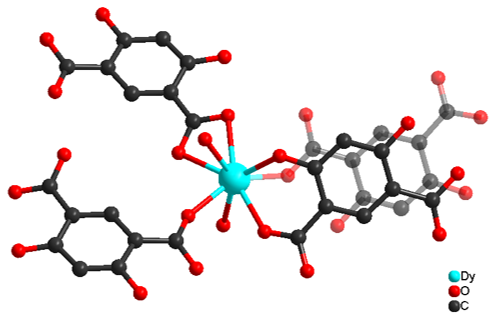


**Supplementary Figure S1.** One Dy(III) center connected by four Hm-dobdc^3−^ ligand and two water molecules.


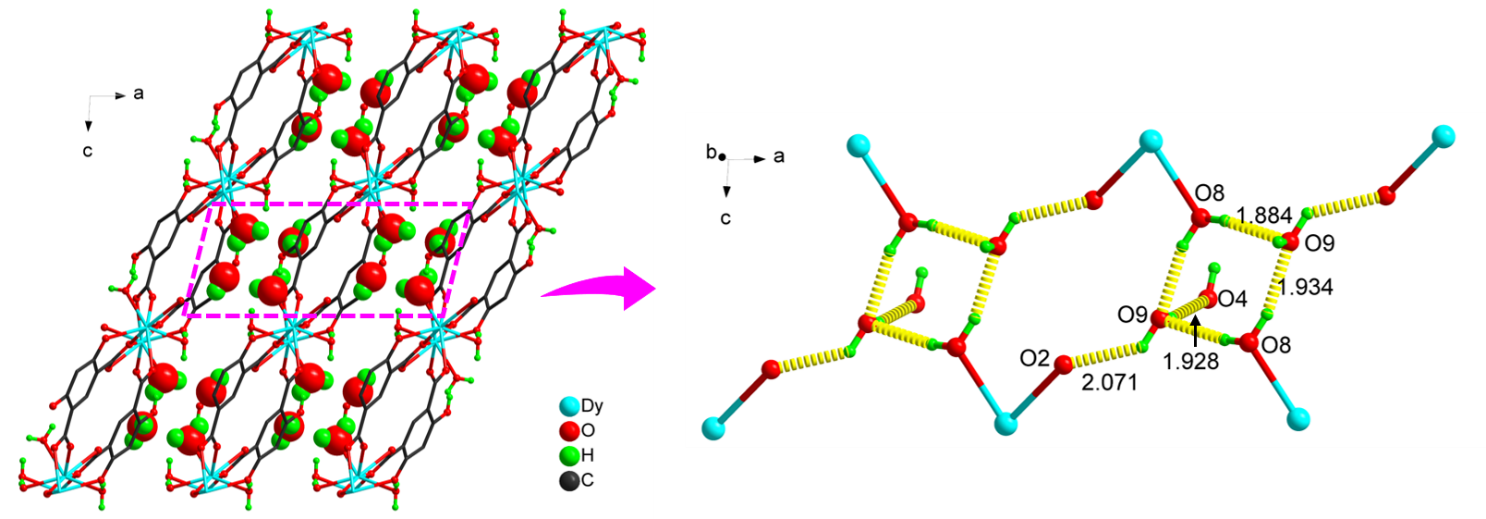


**Supplementary Figure S2.** The coordination environment of the water tetramer located between neighboring layers of **Dy-CP** (lift), and basic hydrogen-bonding building block of tetramer unit (right).


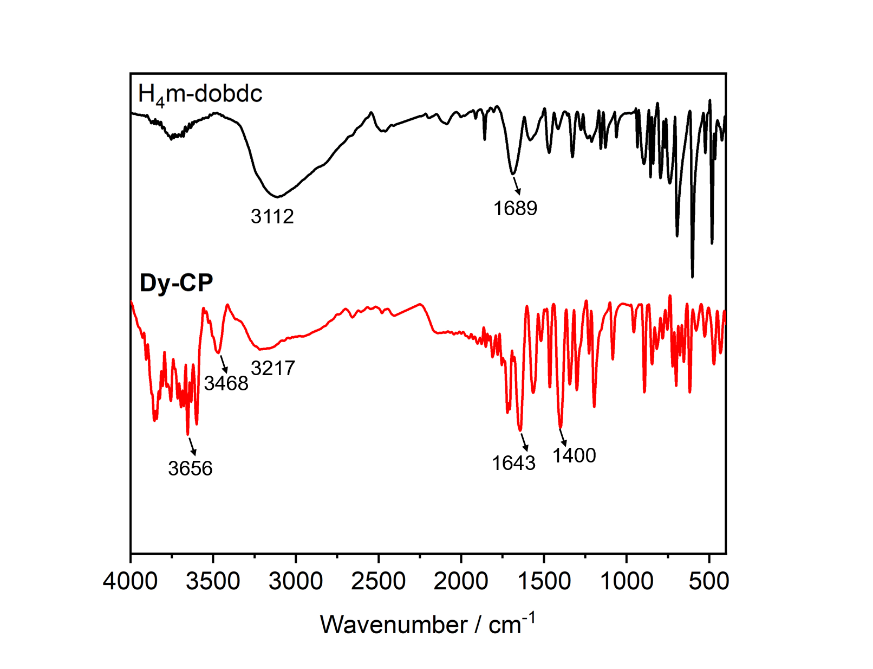


**Supplementary Figure S3.** The FT-IR spectra of H_4_m-dobdc and **Dy-CP**.

~~
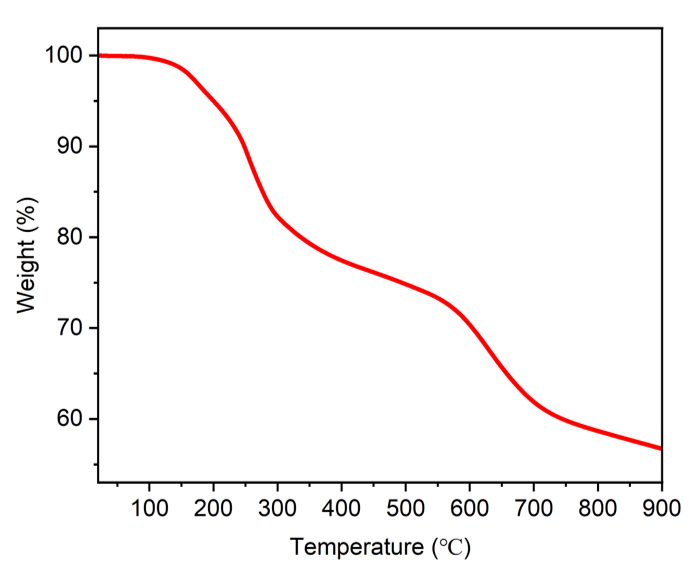
~~

**Supplementary Figure S4.** Thermogravimetric analysis (TGA) curve of **Dy-CP**.


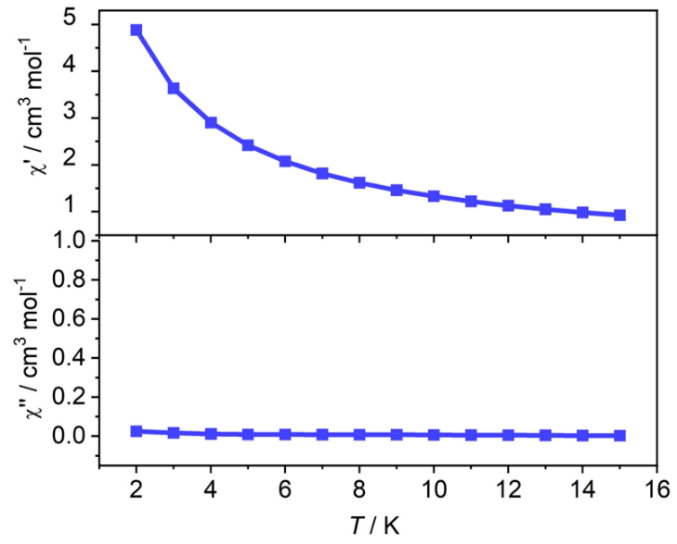


**Supplementary Figure S5.** Temperature-dependent in-phase *χ*′ (up) and out-of-phase *χ*″ (blow) ac susceptibilities for **Dy-CP** measured under zero dc field with an ac frequency of 707 Hz.


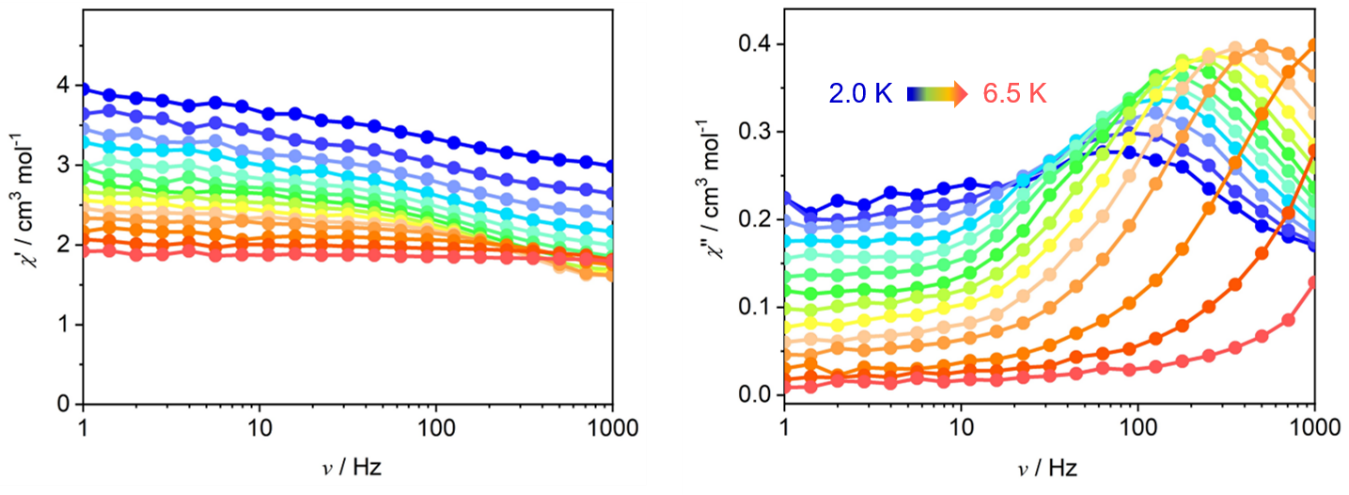


**Supplementary Figure S6.** Frequency-dependent *χ*′ (left) and *χ*″ (right) ac susceptibilities for **Dy-CP** measured in 1.5 kOe dc field.


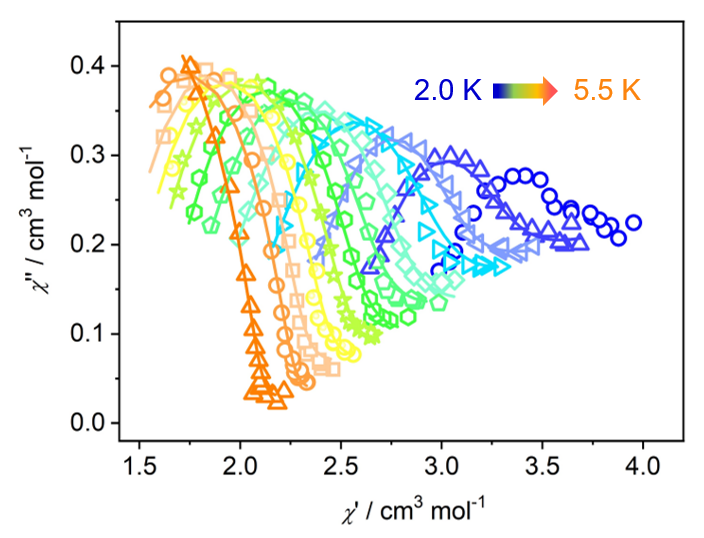


**Supplementary Figure S7.** Cole–Cole plots for **Dy-CP** obtained using the ac susceptibility data at a 1.5 kOe dc field. The solid lines represent the fit to the extended Debye model.


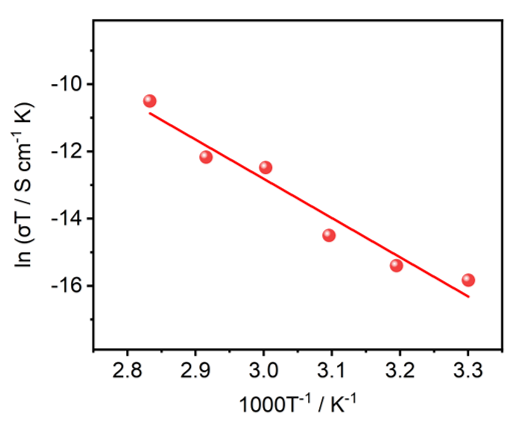


**Supplementary Figure S8.** Plot of ln (*σT*) versus 1000*T*^-1^. The solid red line represents the fitted curve based on the Einstein-Nernst equation, revealing the value of activation energy (*E*_a_)


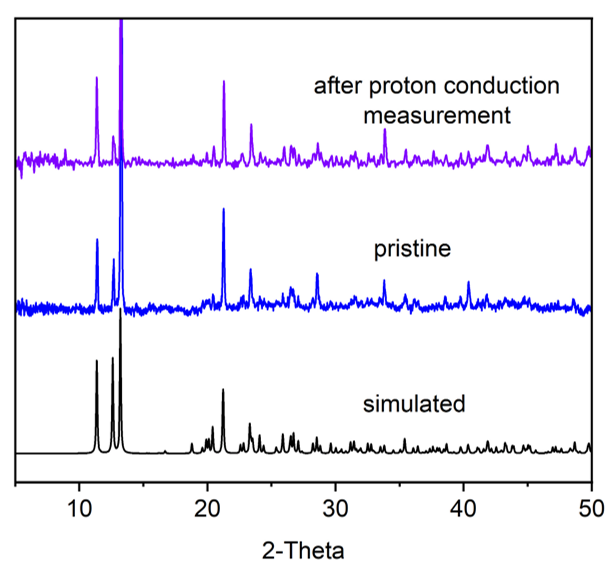


**Supplementary Figure S9.** PXRD patterns of **Dy-CP** for the simulated, synthesized, and sample after proton conduction measurement.


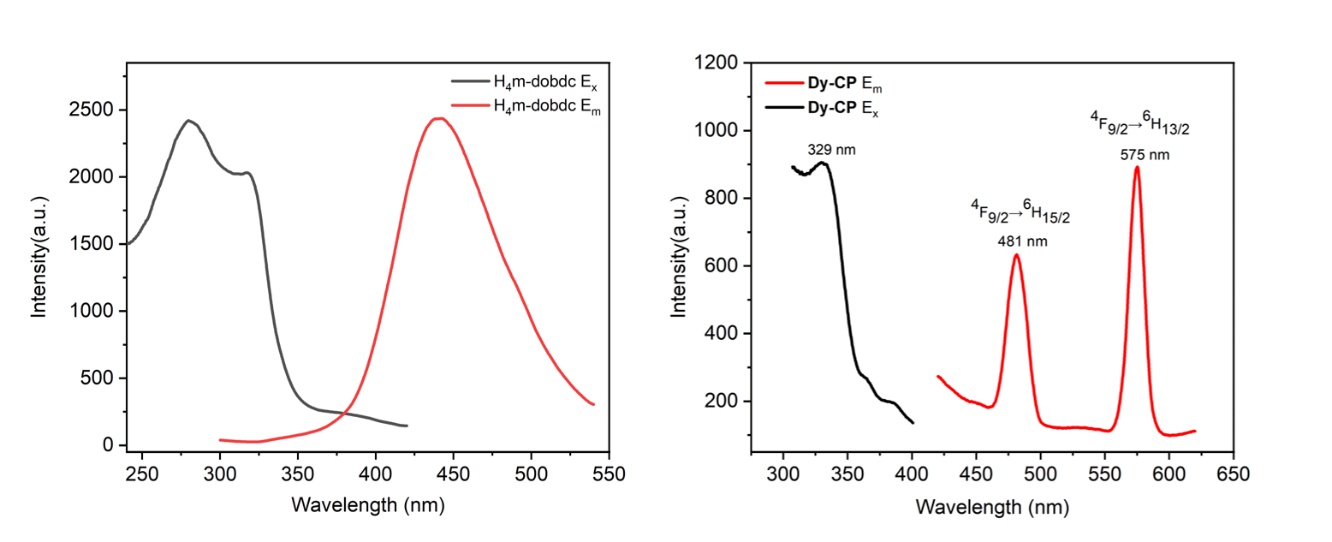


**Supplementary Figure S10.** The solid-state excitation and emission spectra of H_4_m-dobdc (left) and **Dy-CP** (right) at room temperature.

**Supplementary References**

1. Boudreaux, E. A. M., L. N. (1976). Theory and Application of Molecular Paramagnetism; John Wiley & Sons: New York, p1491.
2. Zheng, X., Zhao, X., Huang, J., Yang, H., Wang, Q.,Liu, Y., et al. (2020). Proton conductivity study on three CS/IL@fle-MOF membranes. *Appl. Organomet. Chem.* e5981.
3. *SAINT-Plus,* version 6.02 (1999); Bruker Analytical X-ray System: Madison, WI.
4. Sheldrick, G. M. (1996). *SADABS an empirical absorption correction program;* Bruker Analytical X-ray Systems: Madison, WI.
5. Sheldrick, G. M. (2015). Crystal structure refinement with SHELXL. *Acta Crystallogr. C Struct. Chem.* 71, 3-8.
